# Analysis of Hyperspectral Data to Develop an Approach for Document Images

**DOI:** 10.3390/s23156845

**Published:** 2023-08-01

**Authors:** Zainab Zaman, Saad Bin Ahmed, Muhammad Imran Malik

**Affiliations:** 1 School of Electrical Engineering and Computer Science, National University of Sciences and Technology (NUST), Islamabad 44000, Pakistan; zzaman.msds21seecs@seecs.edu.pk (Z.Z.); malik.imran@seecs.edu.pk (M.I.M.); 2 Department of Computer Science, Faculty of Science and Environmental Studies, Lakehead University, Thunder Bay, ON P7B 5E1, Canada; 3 National Center of Artificial Intelligence, National University of Sciences and Technology (NUST), Islamabad 44000, Pakistan

**Keywords:** hyperspectral imagery, HSI document analysis, spectral unmixing, end-member extraction, abundance estimation, abundance mapping

## Abstract

Hyperspectral data analysis is being utilized as an effective and compelling tool for image processing, providing unprecedented levels of information and insights for various applications. In this manuscript, we have compiled and presented a comprehensive overview of recent advances in hyperspectral data analysis that can provide assistance for the development of customized techniques for hyperspectral document images. We review the fundamental concepts of hyperspectral imaging, discuss various techniques for data acquisition, and examine state-of-the-art approaches to the preprocessing, feature extraction, and classification of hyperspectral data by taking into consideration the complexities of document images. We also explore the possibility of utilizing hyperspectral imaging for addressing critical challenges in document analysis, including document forgery, ink age estimation, and text extraction from degraded or damaged documents. Finally, we discuss the current limitations of hyperspectral imaging and identify future research directions in this rapidly evolving field. Our review provides a valuable resource for researchers and practitioners working on document image processing and highlights the potential of hyperspectral imaging for addressing complex challenges in this domain.

## 1. Introduction

Images composed of varying ratios of Red, Green, and Blue (RGB) are the most widely used form. However, the information provided by this type of image is limited, as only three color wavelengths are used for each pixel. This RGB additive model is not suitable for the segmentation of images containing overlapping texts. In RGB images, we have three-channel information that sometimes may not be adequate in image segmentation. If images are captured with more than three color channels, such as five to ten color channels, using a specialized spectral camera, they are referred to as multi-spectral images. This is because they represent a wider range of channels for each pixel. Multi-spectral images span a wider range of the electromagnetic spectrum, but sometimes a large number of detailed data are needed for each pixel. To capture these data, images with hundreds of wavelengths are taken, providing depth information and resulting in hyperspectral images [1].

Unlike RGB and multi-spectral images, hyperspectral images consist of data with two spatial and one spectral dimension, which can be defined as X×Y×γ, where the spatial dimensions are defined by X and Y representations and the spectral dimension is defined by γ [2]. The spatial dimension of a hyperspectral cube is determined by the size of the individual channels, each of which captures a different narrow wavelength band. The spectral dimension is determined by the number of continuous channels, or spectral bands, that make up the cube. These spectral bands provide a more precise and detailed representation than the two dimensions before it [3]. Given the vast number of data that hyperspectral imagery (HSI) can capture compared to RGB images, it has a variety of applications in complex domains. For instance, HSI is widely used in geo-spatial and satellite imagery, remote sensing, and the spatial modeling of the earth’s surface [4]. HSI imagery also has applications in the field of archaeology [5], forensics [6,7], geophysics [8], document analysis [1,9,10], forgery detection [11], agriculture [12,13], document and ink aging, etc [9,10].

For many years, image processing and computer vision algorithms and techniques have been used for text and document image analysis. Most of these methods are implemented on RGB images. However, a larger proportion of HSI algorithms have been proposed and implemented for Geographic Information Systems (GISs), remote sensing applications, and other geophysical domains. This paper provides an extensive review of existing HSI methods, current libraries, and frameworks and discusses the potential of these algorithms for document image analysis through HSI-based document datasets.

Document image analysis is a swiftly developing field due to technological advancements. Traditional document analysis techniques are based on pattern recognition, optical character recognition, layout analysis, information analysis from historical documents, and automatic document analysis techniques. Most of these techniques are implemented to images acquired from common imagery hardware. One of the drawbacks of this is that RGB images are unable to encode the amount of information that can be stored by hyperspectral images [1]. Hyperspectral imagery has provided dependable and consistent results for applications where images from a traditional camera do not contain enough information to solve the problem at hand. Among all the possible implementations of hyperspectral image analysis algorithms, one of them is in the field of document analysis. In many recent studies, some of the most used methods to acquire, analyze, and utilize data obtained from hyperspectral document images include machine learning, deep learning, auto-encoder decoders architectures, and convolutional neural networks [14].

To implement machine learning for HSI images, the feature-extraction phase is enhanced such that it is capable of capturing feature vectors from high-dimensional input data. These feature representations are then used for feature-to-label mapping, which is used as input for machine learning models to make predictions about the output label of the input data given to it. Most machine learning algorithms that are widely implemented for HSI images are K-Nearest Neighbor (KNN), Maximum-Likelihood Estimation (MLE), Logistic Regression (LR), and Support Vector Machines (SVM) [14].

Deep learning algorithms used for hyperspectral image analysis and processing include artificial neural networks (ANNs), convolutional neural networks (CNNs), Auto-encoder decoders (AEDs), and stacked auto-encoders (SAE). Regarding the implementation of deep learning algorithms for efficient results, it has to be noted that their results heavily depend on the integration of the feature-extraction layer and the classification layer after dimensionality reduction for HSI datasets [14].

Qureshi et al. have discussed hyperspectral document image processing techniques, including some necessary pre-processing steps, namely spike removal, dead pixel removal, and compression. They have discussed that for signature segmentation, document binarization is the initial step, followed by spectral unmixing methods for ink separation. Another method discussed for separate printed text and signatures is to separate both class-image pixels through connected component analysis, followed by using HSI information to extract data for both classes separately, i.e., the signature pixels and other classes present in the sample. HSI’s potential for detecting forged signatures using ink chemical composition analysis through non-invasive methods, thus preserving the document and detecting forgery at the same time, has also been discussed [1].

Kim et al. have explained how the additional bands of HSI images can be helpful in improving the quality of historical artifacts by removing ink bleeds, ink corrosion, and foxing [10]. As different spectra of HSI image pixels store different classes of information, this information can be extracted and separated through spectral unmixing, i.e., end-member extraction and abundance estimation, leading to the separation of unwanted or required classes from the original image. They use the same approach followed by independent component analysis (ICA) to recover lost text. They have worked on enhancing the RGB image of the HSI data and retrieved lost text through gradient map composition. The results from the gradient composition are then checked for enhancement with respect to the corresponding HSI image, and the method explained above is implemented to perform these enhancements. RGB image is again reconstructed from the results that contain all the lost data [10].

Malik et al. have proposed an automated method for signature segmentation in hyperspectral document images to segment the printed text and signature pixels present in the HSI sample through connected component analysis and the processing of local features. Spectral response of pixels of three different classes, i.e., background, text, and signature, are used to extract signature pixels from the three classes of pixels. The proposed method is not affected by color, intensity of ink, and any structural changes to the text as it is based on the spectral response of the pixel class. The discussed method has been tested on 300 images, and results better than the state-of-the-art were acquired [9].

**Figure 1:**
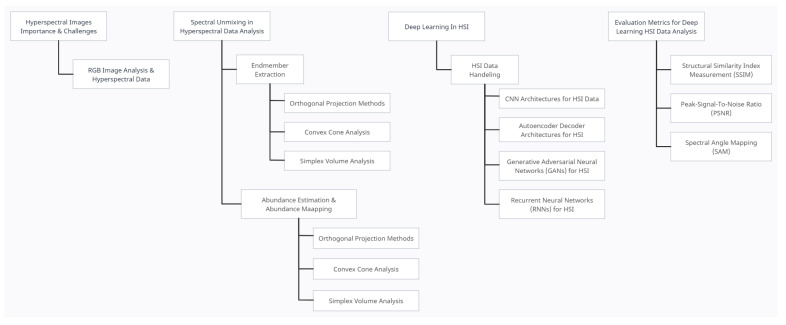
Overview of Survey Contents.

In this paper, we discuss available approaches for hyperspectral data analysis by considering that there is not any specialized approach available for HSI document images. Figure 1 highlights important aspects covered in this paper. The available approaches are designed to deal with the complexities of spatial images. The presented work helps to understand the process that can guide the researchers to contribute to approach development for hyperspectral document image analysis. Section 2 highlights the importance and challenges with HSI data. Section 3 discussed the spectral unmixing methods, whereas the role of deep learning in HSI is discussed in Section 4. Various evaluation metrics used in the literature to assess deep learning performance on HSI data are elaborated in Section 5. Future aspects are discussed in Section 6.

## 2. Hyperspectral Images: Importance and Challenges

Among numerous challenges while processing and analyzing hyperspectral images, the most likely challenge to face is handling a large number of channels simultaneously. As discussed earlier, unlike RGB images, hyperspectral images consist of *n* hundreds of channels, thus making each pixel an array of *n* elements. Each channel and thus each pixel encodes information about various components of the sample image. Loading the image, pre-processing, removing noise, and analyzing information on the channel level or per pixel per channel level can prove to be a complicated task. Implementations of HSI algorithms and methods as a solution to a variety of use cases involving datasets and hyperspectral images of different natures are listed in Table 1.

In applications where classification, detection, and segmentation are the task at hand and require a high level of precision, HSI is used because of its characteristic of containing both spectral and spatial information at the pixel level. HSI imagery captures data at different wavelengths, making them more information-rich than traditional RGB or multi-spectral images. The in-depth information represented by hyperspectral images can be helpful in detecting different textures, chemicals, inks, etc., at pixel level [15].

Apart from these applications, they can be employed in document image analysis for signature segmentation, document aging through ink spectra analysis, forgery detection, and historical document analysis through the implementation of various techniques such as ink mismatch detection through analysis of spectral signatures and HSI coupled with chemometric techniques for near-infrared (NIR) and infrared bands in the HSI cube [1,9,10]. The characteristics of RGB and hyperspectral images vary in different respects, as elaborated below.

### RGB Image Analysis and Hyperspectral Data

Digital images are obtained from scanners, digital cameras, and computers. This process involves converting real-world images into data consisting of rows and columns of various color samples, which are calculated from the original image using tricolor channels.

The digital images are depicted through pixels. Pixels are a fundamental unit that make an image digital. The digital image’s dimensions are represented as pixels, which are important to determine prior to performing any experiment. In digital images, pixels are aligned in rows and columns representing color values in the tiny squares, which are added together to show a bigger picture of an image. The alignment of pixels in digital images is usually overlooked by the human brain, but this complicated work is performed by the computer itself. On each tiny square of an image, there is a numeric number representing an RGB data value. In general, each pixel is composed of the proportion of three color wavelengths. It is important to mention that each color in RGB has 256 shades, and one pixel separately contains different shades of R, G, and B. In most images, three-channel color (i.e., RGB) information is sufficient to produce useful data from images. But in special circumstances where segmentation is required in overlapping regions, the three channels fail to assist. In such situations, we look for more pixel data.

**Figure 2:**
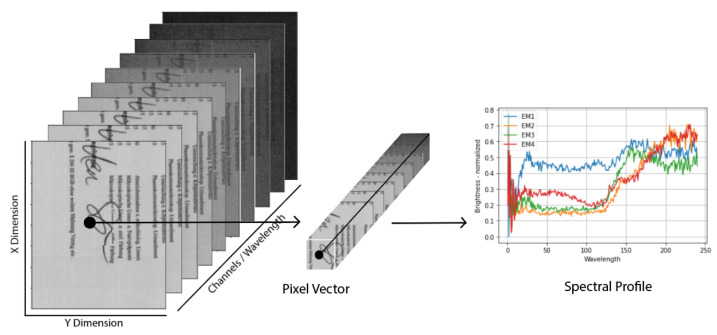
HSI Data Cube.

In RGB images, we have three channels/spectral information, but in hyperspectral images, there are hundreds of channels. Each pixel represents spectral information that can help in segmenting two different contents of an image in overlapping regions. In hyperspectral images, HSI sensors capture data ranging within a broader spectrum than RGB and containing a larger number of channels, thus capturing greater information embedded within them. Data captured through HSI sensors and imaging devices are in the form of an HSI image cube comprising both spatial and spectral information, as shown in Figure 2. Because granularities that can be captured through HSI imaging techniques, they have a broader scope of application. Among the most important applications of HSI is its application to document image analysis for signature verification, forgery detection, historical documents analysis and restoration, etc. Various hyperspectral analysis techniques exist for this purpose.

A majority of existing methods for hyperspectral images have their implementations in the GIS domain. These implementations cannot be directly implemented on hyperspectral document images due to limitations in the nature of the particular problem and dataset.

**Figure 3:**
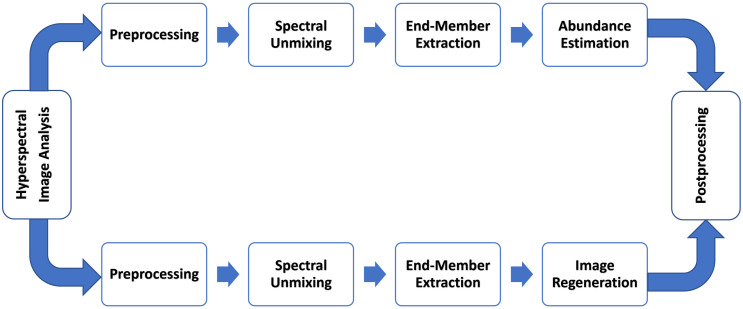
HSI Workflow.

Hyperspectral document image analysis is performed through two workflows, as depicted in Figure 3. The backbone of all existing HSI methods is the method known as spectral unmixing. Spectral unmixing is basically the process of breaking down the captured spectra into their respective constituents, known as “End-Members”, and their corresponding fractions, known as “Abundances” [16]. Spectral unmixing gives the relative abundance of the relevant components that make up the hyperspectral scene. HSI document images can be analyzed using end-member extraction and also through abundance maps or a combination of both.

In this paper, we are investigating the available end-member-extraction methods and abundance-estimation methods. The available methods are specifically designed for terrain image analysis, but their potential can be explored in HSI-based document image analysis and hence can be useful in proposing an approach for hyperspectral document images.

## 3. Spectral Unmixing in Hyperspectral Data Analysis

In hyperspectral data analysis, spectral unmixing serves the purpose of extracting valuable information about the composition and spatial distribution of materials within a scene captured by a hyperspectral imaging system. Hyperspectral data contain detailed spectral information for each pixel, spanning a wide range of contiguous and narrow spectral bands. However, in real-world scenarios, the observed spectra often represent a mixture of multiple materials present within a pixel, making it difficult to directly interpret the data.

The goal of spectral unmixing is to unravel this spectral mixture and identify the individual components, known as end members, that contribute to the observed spectra. Each end member represents a pure spectral signature associated with a specific material or substance of interest.

By decomposing the mixed spectra into their constituent end members, spectral unmixing enables researchers and analysts to estimate the abundance or proportion of each end member within each pixel. This abundance information provides insights into the presence and spatial distribution of different materials in the scene. With spectral unmixing, it becomes possible to map and quantify the distribution of specific materials or substances, such as different vegetation types, minerals, pollutants, or human-made objects. This information can be utilized in various applications, including remote sensing, environmental monitoring, geology, agriculture, and target detection, and overlapping text analysis.

The process of spectral unmixing involves the extraction of endmembers from the hyperspectral data and the estimation of their abundances within each pixel. This allows for the generation of abundance maps, which depict the spatial distribution of individual materials, enabling a detailed analysis of the scene.

An enormous number of existing unmixing strategies perform end member extraction and abundance estimation separately as a two-step process that initially estimates the endmembers through one of the multiple methods presented in various studies and then computes their corresponding abundances through abundance-estimation procedures with or without human interaction [17].

Two major approaches exist for spectral unmixing are,

1. *Full Unmixing Problem (FUP):* In the absence of any information regarding endmembers or abundances, the unmixing process implemented is known as FUP.
2. *Abundance Estimation Problem (AEP):* Implementing end-member extraction and abundance estimation with prior information about the end members is known as AEP.

Various approaches for spectral unmixing are presented in the literature [17,18], and most of them have their basis on the Linear Mixing Model (LMM) [19] that works on the supposition that the spectrum of a mixed pixel is linear combination of pure spectra of the components present in a specific pixel weighted by their fractional coverage with respect to other components present in that pixel [20]. The basic concept of LMM is that there is no multiple scattering between end members; i.e., incident light interacts with only one end member at a given time, which has been explained by the formula in [20],

(1) $$y=\sum_{i=1}^{n}x_{i}m_{i}+c$$

(2) y=Mx+c

where the matrix of end-members is defined by *M* and the spectral signature of the $i^{th}$ end-member $m_{i}$ is defined by *M*$\in {ℜ}_{+}^{m+n}$, *x* is the vector of abundances such that *x* $\in {ℜ}_{+}^{n}$ and x≥0 & $\sum^{n}x_{i}\leq 1$ or $\sum^{n}x_{i}=1$. *y* is the measured pixel spectrum such that *y*$\in {ℜ}^{m}$, *c* is the noise vector, the number of end-members is given by *n,* and the number of spectral channels of the sensor is defined by *m* [17,21,22].

### 3.1. End-Member Extraction

Pixels in multi-spectral and hyperspectral images are composed of various materials, each of which has a unique spectral signature term as the end member. Each end member has a unique spectral signature that segments it from the rest.

End-member extraction is one of the processes of spectral unmixing. Unlike abundance estimation, the end member extraction may or may not be a fully automated process. Various algorithms are used for end-member extraction, falling under three categories, i.e., orthogonal projection methods, convex cone analysis, and simplex volume analysis. Among these three categories, the most popular is orthogonal projection methods, which include the Pixel Purity Index (PPI), Fast Iterative Pixel Purity Index (FIPPI), Automatic Target Generation Process (ATGP), and N-FINDR.

The end-member extraction process comprises a list of steps that include end-member finding, end-member extraction, end-member determination, end-member selection, end-member optimization, end-member verification, and end-member identification. The method to be implemented for end-member extraction depends on the nature of the problem and the available dataset without any prior knowledge of the existence of potential end-members in the pixels. However, end-member determination is the process of determining end-members from a set of candidate points that may have potential end-members. Finding the optimum collection of end-members to characterize the data is the goal of end-member optimization, and recognizing end-members from the data without the use of a spectral library is the goal of end-member identification [23]. End members obtained through the implementation of these methods are evaluated based on variability and discriminability. The implementation of the available end-member-extraction algorithms on diverse datasets is listed in Table 1, and details about each class of algorithms and their available methods are explained below.

#### 3.1.1. Orthogonal Projection Methods

Orthogonal projection is a means of representing three-dimensional objects in two dimension planes [24]. It is a form of projection technique in which all the projection lines are orthogonal, i.e., at an angle 90 degrees to the projection plane.

The orthogonal projection for finding end-members is one of the earliest methods implemented for this purpose. Some of the most used algorithms for end-member extraction fall under this category, including Pixel Purity Index (PPI), Automatic Target Generation Process (ATGP), and Vertex Component Analysis (VCA).

##### Pixel Purity Index (PPI)

Due to its incorporation into the ENVI software (5.6.3) suite https://www.l3harrisgeospatial.com/docs/gettingstartedwithenvi.html (Accessed on 13 March 2023), the Pixel Purity Index (PPI) is one of the frequently utilized techniques for end-member extraction. After dimensionality reduction and noise whitening with the Maximum Noise Fraction (MNF) transformation, the method iteratively projects data to random unit vectors to determine pixel purity [18].

The PPI algorithm works in a series of four steps to find end members. The first step is the initialization process, where the number of dimensions to be retained, *d*, is determined, and the dimensionality of the dataset is then reduced to these *d* dimensions through dimensionality reduction followed by the generation of a random set of unit vectors *U* explained through the following expression,

(3) $${\left\{Vector_{u}\right\}}_{u=1}^{U}$$

where *U* is supposed to be a very large positive integer. Once the above steps are completed, the PPI count is calculated for each $Vector_{u}$ by projecting all data samples orthogonally onto $Vector_{u}$ to find a set of extra points for that particular vector denoted by $V_{extrema}\left(Vector_{u}\right)$. To calculate the PPI count, an indicator function $F_{V}\left(e\right)$ is defined for these sets of extrema points $V_{extrema}\left(Vector_{u}\right)$, which calculates the PPI count for each sample vector *e*. To find end-members, these PPI counts for the dataset are thresholded on a selected value and all the PPI counts greater or equal to that defined threshold make up the end-members for that dataset. However, PPI produces inconsistent results because of using randomly generated end-members and requires a trained analyst to manually select the best possible set of end-members [23]. As a suggested enhancement to PPI, the Fast Iterative Pixel Purity Index (FIPPI) calculates the number of end-members through virtual dimensionality until it reaches the final set of end members after iteratively producing an adequate set of beginning end-members [18].

**Table 1:** End-Member Extraction Algorithm Implementations In the Available Literature.

Method	Nature Of Dataset	Sample	Studies
*Orthogonal Projection Methods*			
*Pixel Purity Method (PPI)*	AVIRIS Cuprite Dataset	Satellite Images	Guo et al. [25]
	AVIRIS Cuprite Dataset	Satellite Images	Gu et al. [26]
	Real hyperspectral image scene collected by Hyperspectral Digital Imagery Collection Experiments (HYDICE)	Urban Images	Chang et al. [27]
	AVIRIS Cuprite Dataset	Satellite Images	Sanchez et al. [28]
	AVIRIS Cuprite Dataset	Satellite Images	Gonzalez et al. [29]
	AVIRIS Cuprite Dataset	Satellite Images	Wu et al. [30]
	AVIRIS Cuprite Dataset	Satellite Images	Valancia et al. [31]
*Automatic Target Generation Process (ATGP)*	HSI images captured via the HyMap airborne hyperspectral imaging sensor with coverage areas of 2.0 km^2^ in Cooke City town, MT, USA	Urban Images	Khoshboresh et al. [32]
	HYDICE 15-panel scene and HYDICE urban scene	Urban Images	Chang et al. [33]
	HSI data collected by NASA’s AVIRIS instrument over the World Trade Center (WTC) in New York, five days after the terrorist attacks that collapsed the two main towers	Urban Images	Sierra-Pajuelo et al. [34]
	HSI data collected by NASA’s AVIRIS instrument over the World Trade Center (WTC) in New York, five days after the terrorist attacks that collapsed the two main towers	Urban Images	Paz et al. [35]
	HSI CD Dataset	Urban Images	Yadav et al. [36]
	HYDICE Dataset	Urban Images	Chang et al. [37]
	Custom dataset collected via the hyperion sensor over the mangrove habitats of Henry Island, West Bengal	Urban Images	Chakravortty et al. [38]
*Convex Cone Analysis (CCA)*			
*Convex Cone Analysis (CCA)*	WorldView-3 (WV3) Very High Resolution (VHR) satellite frame, HSI signatures of urban seismic rubble acquired through an ASD FieldSpec Pro hand-held radiometer, HSI signatures of uncolored typical cover and Pixel Digital Terrain Model (DTM) derived from LiDAR data about Amatrice	Urban Images	Pollino et al. [39]
	Landstat Dataset	Satellite Images	Milewski et al. [40]
	Custom dataset of ASTER Level 1T (L1T) data acquired in August 2003	Rock & Mineral Images	Esmaeili et al. [41]
	Custom dataset collected through HSI microscope setup using Zeiss Axiovert 100 inverted microscope	Blood Images	Lee et al. [42]
	MODIS NDVI Data.	Knight et al. [43]	
	cloud-free satellite data which was derived from the United State Geological Survey (USGS)	Satellite Images	Singh et al. [44]
	Custom dataset collected through HSI sensor on UAS platform by the Carinthia University of Applied Sciences (CUAS), Austria	Time Series & Urban Images	Milewski et al. [40]
*Simplex Volume Analysis (SVA)*			
*NFINDR*	Geostationary Ocean Color Imager (GOCI) dataset and HJ-1B dataset	Geostationary Ocean Satellite Images	Tao et al. [45]
	AVIRIS Cuprite Dataset	Satellite Images	Xiong et al. [46]
	AVIRIS Cuprite Dataset	Satellite Images	Ji et al. [47]
	Airborne Imaging Spectrometer for Application (AISA) and Compact Airborne Spectrographic Imager (CASI) dataset	Airborne Images	Song et al. [48]
	AVIRIS Cuprite Dataset	Satellite Images	Quirita et al. [49]

##### Automatic Target Generation Process (ATGP)

**Figure 4:**
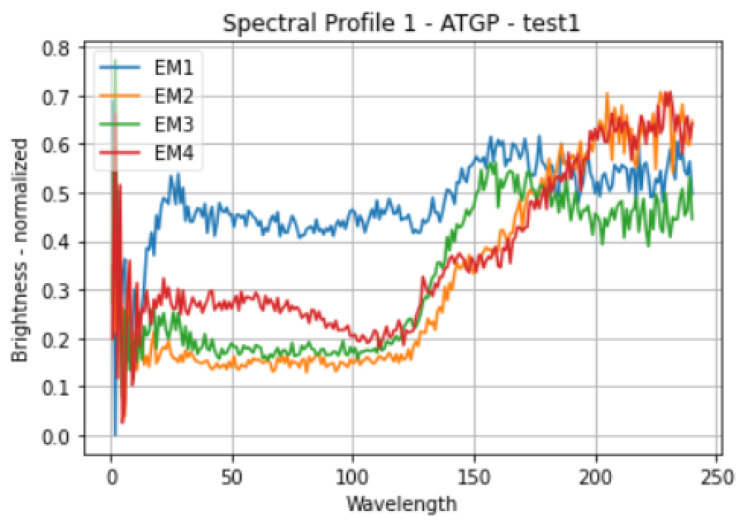
Spectral profile of four end-members generated through the ATGP method.

In contrast to PPI, ATGP finds the data vectors that are at the ends of specially constructed projection vectors because these vectors have maximal orthogonal projection and are thus more likely to be end members. The limitations of PPI are addressed by ATGP, although number of end members must be explicitly designated as a stopping condition for the algorithm [23]. The spectral profiles of four end-members generated through the ATGP method are shown in Figure 4.

##### Vertex Component Analysis (VCA)

The Automatic Target Generation Process (ATGP) and Vertex Component Analysis (VCA) can be thought of as variants of each other, with the main differences being the use of a Gaussian random number generator to generate the initial set of end-members and the fact that VCA has a restricted search region in the first quadrant (controlled by non-negative abundance fractions), making it incomparable to ATGP. Because of these issues, ATGP is chosen over VCA [23].

#### 3.1.2. Convex Cone Analysis (CCA)

The main goal of convex cone analysis is to determine how to locate end-members by computing the convex cone’s volume through convex cone projections (CCPs) and determining the maximal convex cone volume (CCV), which is comparable to determining the CCP’s maximal simplex volume (SV) in a hyperplane through the use of NFINDR, which is covered in Section 3.1.3. By assuming that the vertices of the formed cone when the hyperplane cuts through the convex cone in the data space are orthogonal to the hyperplane, convex cone volume analysis (CCVA) essentially serves as the foundation for simplex volume analysis (SVA) techniques. CCV is determined as the product of the height of the simplex and the orthogonally projected simplex on the same hyperplane, which is the orthogonal distance of the convex cone to the hyperplane. The same procedure is used to calculate SV through NFINDR, which results in the discovery of the OP with the highest volume in the original data space, which is identical to the simplex with the highest volume in that hyperplane [23]. CCA and CCVA form the basis of simplex volume analysis (SVA) methods discussed in Section 3.1.3.

#### 3.1.3. Simplex Volume Analysis (SVA)

In optimization problem solving, a simplex method is a technique that utilizes slack and pivot variables to calculate the best possible solution. In the context of convex cone analysis (CCA) described earlier, it also falls under a category of analysis techniques based on convex conevolume (CCV). Convex cone projection (CCP) is used in these approaches to construct a simplex by projecting the recognized convex cone onto a hyperplane. Both the Abundance Sum-to-One Constraint (ASC) and the Abundance Non-negativity Constraint (ANC) are satisfied by the simplex created by CCP [23].

Using NFINDR, end-members are found by locating the convex cone in the data space with the largest CCV. Finding the simplex created by CCP with the largest simplex volume on the hyperplane is analogous to this procedure.

##### NFINDR

NFINDR is an early method used for geometric end-member induction. It operates by starting with a random set of pixel spectra from an image as the initial end-member set. It then evaluates each pixel separately from the initial set to determine if it can potentially replace one of the end members. This process expands the volume of the simplex, allowing pixel spectra to be considered as end-members. The algorithm continues until there are no more viable candidate replacements. Finally, it reduces the orthogonal subspace projection to N-1 dimensions, where N represents the number of end members [18].

### 3.2. Abundance Estimation and Abundance Mapping

**Figure 5:**
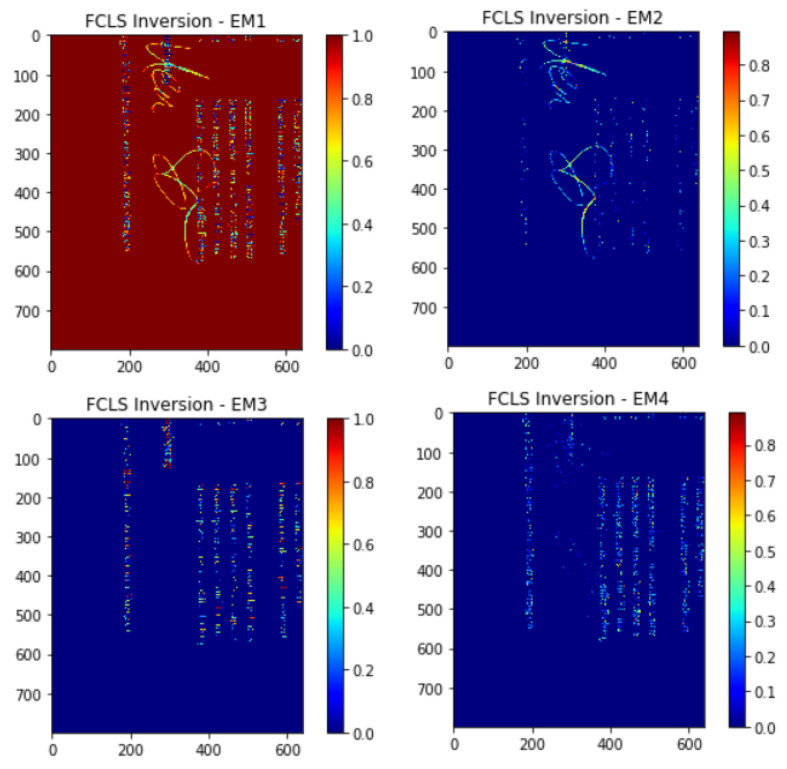
Abundance maps of end-members are shown in Figure 4 produced through the FCLS method.

The abundance-estimate techniques are then put to use to determine the abundance fractions of the extracted end-members in a pixel of the hyperspectral image after end-members have been discovered and extracted using any of the aforementioned algorithms as shown in Figure 5. Prior to performing an abundance estimate, the numbers of end-members are already known. Without the assistance of a human, these abundance-estimate methods determine the percentage of each end-member in a certain hyperspectral picture pixel. The abundance fractions shown in Figure 5 are generated through our initial experimentation.

Table 2 provides a list of the existing Python implementations of the end-member extraction and abundance estimate techniques described in the literature, along with web links to corresponding Python libraries.

Least squared methods are a class of abundance-estimation algorithms that fall under inversion methods [19] with the key constraints being non-negativity—i.e., decision variables would be positive irrespective of the maximization or minimization problem—and full-additivity, i.e., each variable involved contributes towards the final outcome. As discussed in Equation (1), *c* is the noise vector, which can be interpreted as a measurement or model error, and *M* is the matrix of end-members $M\;=\;\{m_{1},\;m_{2},\;m_{3},\;...,\;m_{i}\}$, and least squared methods [16,56] can be helpful in implementation of the abundance fractions $x\;=\;\{x_{1},\;x_{2},\;x_{3},\;...,\;x_{i}\}$ to solve for non-negativity and full-additivity constraints as mentioned earlier. As a distance-minimization problem (DMP), the abundance estimation problem is tackled using least squared methods (LSMs) to reduce the distance between the processed spectral estimate and the original spectral response [16]. A number of classes of LSM algorithms exist for this purpose, which includes Unconstrained Least Squared (ULS) Algorithms, Non-Negative Least Square (NNLS) Algorithms, Unsupervised Non-Negativity Constrained Least Squared (UNCLS) method, Unsupervised Fully Constrained Least Squared (UFCLS) method, Image Space Reconstruction Algorithm (ISRA), and HSI Abundance Estimator Toolbox (HABET). Each one is briefly explained as follows.

#### 3.2.1. Unconstrained Least Squared Methods (ULSs)

ULS solutions consider that there are *j* end-members in an image, i.e., $a_{1},\;a_{2},\;...,\;a_{j}$, and any image pixel vector is a mixture of *j,* with abundance fractions being $\phi_{1},\;\phi_{2},\;...,\;\phi_{j},$ with $\phi_{i}$ corresponding to the abundance fraction of the $i^{th}$ end-member and is explained on the basis of Equation (2) as

(4) I=Aϕ+n

where *A* is the end-member matrix, ϕ is the vector of abundance fractions of end-members $a_{1},a_{2},...,a_{j}$, which are defined as [16,23]

(5) $$y=\sum_{i=1}^{j}\phi_{i}=1$$

with two basic constraints for ϕ being ϕ≥0, as per Equation (3) $\phi_{i}\geq 0$ ∀ 1≤i≤j.

#### 3.2.2. Non-Negative Least Squares (NNLS)

The approach implemented for NNLS is to iteratively calculate the fractional abundance values $\phi_{1},\;\phi_{2},\;...,\;\phi_{j}$, and out of those values, the least squares of the coefficients with negative values are computed with respect to the corresponding values of $a_{1},\;a_{2},\;...,\;a_{j}$ in the end-member matrix *A* [21]. The coefficients of *A* used to calculate the least squares at a given iteration make up the active set ψ and the set of coefficients that do not contribute to the least squares solution at a given iteration make up the inactive set Γ. Based on this approach, the NNLS is also referred to as the active set strategy. The NNLS is an implementation of the least squares problem with linear inequality constraints, defined as

(6) min||Ax−b||,givenGx>h

where $A\in {ℜ}^{mxn},b\in {ℜ}^{m},G\in {ℜ}^{mxn},G\in {ℜ}^{m}$ [17].

Based on the non-negativity least squared (NNLS) method, there is another method known as the unsupervised fully constrained least least squared (UNCLS), method which imposes the following abundance non-negativity constraints ϕ≥0 ∀ $\phi_{i}\geq 0$ on *I*, defined by Equation (3) [23].

#### 3.2.3. Unsupervised Fully Constrained Least Squared Method (UFCLS)

UFCLS is a technique where Fully Constrained Least Squares (FCLS) is a partial abundance-constrained orthogonal subspace-projection technique that implements $\sum_{i=1}^{j}\phi_{i}=1$ & ϕ≥0, i.e., $\phi_{i}\geq 0$, on the linear mixing model [23]. FCLS therefore requires comprehensive knowledge of the signature matrix to implement it when no information about the signature matrix is known. An unsupervised method is implemented first to compute the required information for the FCLS. Two of the commonly used methods include the nearest neighbor method and ATGP.

UFCLS is implemented by first selecting a pure pixel $p_{0}$ as the initial signature, which may be a single signature or a set of signatures depending upon the problem at hand. After selecting the pure signature, a pixel vector is mostly distinct from $p_{0}$; i.e., the largest least squared error (LSE) between $p_{0}$ and itself is selected and marked as $p_{1}$. This then provides the material matrix *P = [$p_{0}$$p_{1}$]* required to run FCLS, which is then executed to obtain abundance fractions $\phi_{0}^{\left(1\right)}\left(a\right)$ and $\phi_{1}^{\left(1\right)}\left(a\right)$ for each pixel vector *a* for $p_{0}$ and *$p_{1}$,* respectively. For any picture pixel vectors, the LSE between *r* and its linear mixture may be determined using these abundance predictions. The third pure pixel signature would subsequently be chosen as the one with the biggest LSE. Until the LSE is sufficiently small or falls below a certain threshold, these repetitions are repeated. The UFCLS algorithm is implemented in the manner described above [57]. Table 3 summarizes the work presented in the field of HSI image analysis.

#### 3.2.4. Image Space Reconstruction Algorithm (ISRA)

The Image Space Reconstruction Algorithm is from the “Positive Constraint Only Algorithm” class. ISRA works on the constraint that if any value of $\phi_{1},\;\phi_{2},\;...,\;\phi_{j}$ is positive, it remains positive, and if any coefficient is zero, it remains zero. ISRA assumes that ϕ would always be a non-negative initial value and the abundance matrix *A* would be a non-negative abundance matrix. Until the error falls below the specified error threshold δ or until the estimated abundances coefficient has less distance from the previously calculated abundance coefficient, the method would repeat. ISRA converges to the solution if the problem at hand belongs to a consistent system and if the problem at hand belongs to an inconsistent system and contains noise. The ISRA converges to the solution that is closest to the estimated system solution [17].

#### 3.2.5. HSI Abundance Estimator Toolbox (HABET)

HABET is a graphical-user-interface-based HSI abundance estimator toolbox for ENVI. ENVI image processing and analysis software provides a complete set of tools for processing hyperspectral data that includes spatial mapping, spectral unmixing, and classification commonly used in GIS [66].

HABET is a GUI-based abundance estimator providing access to hyperspectral image analysis and processing methods for opening HSI files, displaying HSI files, implementing abundance estimation methods, and generating abundance maps. HABET toolbox works by obtaining HSI data from memory that is selected by the user for processing along with the end-members data, which are then passed to the selected abundance-estimation routines out of the available methods, i.e., ISRA, NNLS, non-negative sum less than or equal to one (NNSLO), and non-negative sum to one (NNSTO). The toolbox then provides the abundance maps corresponding to the HSI and end-member data provided by the user [17].

## 4. Deep Learning in HSI

Recent advancements in HSI image analysis due to its depth coordinates have increased the importance of hyperspectral data to manifolds. However, due to the large dimensionality of the input data, restricted availability, and high intra-class variability, HSI image analysis deep learning (DL) architectures show complexity [67].

The following are the characteristics that deep learning architecture must exhibit [67].

1. With or without manually constructed feature extraction methods, deep learning networks may extract linear and non-linear characteristics from raw data.
2. Deep learning architectures can handle various forms of data; for example, in the case of HSI datasets, they can handle spectral and spatial data separately and simultaneously as well.
3. Depending upon the nature of the problem and the type of available dataset, the choice for the architecture and implementation of the learning strategy varies.
4. Due to the presence of various processing techniques such as batch partition [68], high-performance computing (HPC) [69], and parallel computing [70,71], deep learning architectures can better scale when dealing with large amounts of data.

Deep learning architectures’ effectiveness is dependent on the automatic and hierarchical learning process from the spatial domains of the data, as well as the spectral domains in the case of HSI datasets. Given that the deep learning model contains a high number of parameters, a relatively large dataset is required for training so that we can avoid the overfitting of the learning model. A dataset in such a case comprises hundreds of thousands of samples [72]. As discussed previously, features extracted from HSI data are of the dimensions $X\in {ℜ}^{n_{1},n_{2},n_{b}ands}$ and are composed of their two spatial components, $n_{1}$ and $n_{2}$, and their spectral domain $n_{bands}$. Although these features can be processed through ML models, DNNs stand out by adapting to the processing of such types of features through deep neural net architectures [67]; however, while implementing DNNs for HSI data the following challenges need to be handled.

**Figure 6:**
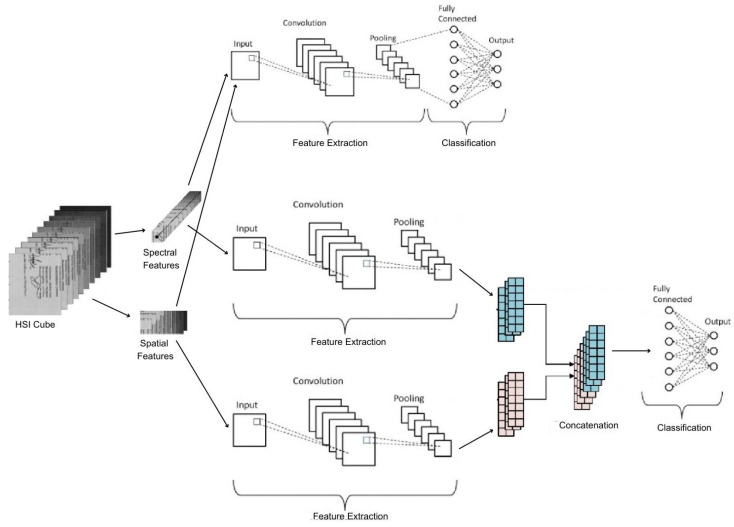
Deep Learning Pipeline for HSI Data.

1. Due to their propensity to overfit if the training set only contains a few training samples, DNNs are inefficient at generalizing the distribution of HSI data. The DNN architecture being implemented is more prone to overfitting, necessitating changes during the training phase, limited generalization, and poor performance on the test set in the case of HSI datasets because of the high dimension and sparse training examples.
2. Due to the curse of dimensionality, DNN architectures for HSI are computationally expensive and memory-intensive.
3. Deeper networks with more parameters make training, optimization, and convergence more challenging and could result in several local minima.
4. With the training process being a black box and the number of parameters for HSI, although various visualization processes can be implemented to visualize output at every layer, implementing optimization decisions and implementing more significant and interpretable filters is a tedious job.
  Due to the above-mentioned challenges and issues, researchers and developers have attempted to implement DNNs as shown in Figure 6, choosing the best-fit learning strategies and optimization processes that are best suited for HSI datasets while maintaining computational efficiency [67].

A major portion of work implemented through HSI datasets is related to the remote sensing field, with all methods and functions being dedicated to the remote sensing or geo-spatial field specifically, with very little work being implemented in other domains such as forensics, document analysis, biomedical, target detection, anomaly detection, data enhancement, etc. [72].

### 4.1. HSI Data Handling

In the case of HSI datasets, most available datasets comprise few hundred samples with relatively higher dimensionality, which may or may not be labeled, leading to the issue of sparsity because of the presence of a lesser number of samples that contribute to training, and the inability of the model to generalize training on such data may lead to overfitting. Such issues may be resolved by implementing unsupervised techniques, which are specialized to learn the network in the absence of labeled data.

By extracting spectral signatures from either individual pixels or groups of pixels, DL architectures focus on HSI data work by extracting the data in a pixel-by-pixel fashion. For instance, an object-detection approach must be constructed before obtaining object-wise spectral signatures. To resolve the curse of dimensionality issue and the redundancy issues that come with dimensionality reduction methods like PCA [73], independent component analysis (ICA) [74] and stacked auto-encoders [75] have been implemented to extract the problem-relevant features instead of handling the large number of dimensions of HSI datasets followed by spatial processing or 3D-patch processing [72].

The number of layers, kinds of layers (such as convolution, pooling, activation, softmax layers, etc.), activation function, problem to be solved, and learning procedures are the factors that determine how efficient and successful a deep neural network is. The most popular deep learning architectures incorporate characteristics such as space-invariance [76], robustness, and deformation; require fewer data; and implement powerful regularization techniques [77].

### 4.2. Deep Neural Network (DNN) Architecture

The perceptron is the fundamental building block of every neural network, and it is responsible for performing computation from single-layer to complex deep learning architectures. Neural networks are aggregations of neurons taking inputs in the form of either an input to the whole network from the input layer or an input in the form of the output of the preceding layer. A neuron takes all inputs, aggregates them, and produces the output, which may be passed to another neuron in the proceeding layer, which may be a component of the activation layer, a fully connected layer, a convolution layer, or a pooling layer. The depth of the network, type of activation used, number of pooling layers, and type of layers depend on the nature of the dataset to be processed [67].

Multiple deep learning architectures have been implemented to handle and process hyperspectral data, some of which are based on the CNN [78], autoencoder–decoder [79], generative adversarial neural networks [80], or the recurrent neural networks [81]. Implementations of a few of these architectures are detailed in Table 4 and explained as follows.

#### 4.2.1. CNN Architectures for HSI Data

CNNs have become an important component in hyperspectral image analysis. Hyperspectral imaging is a powerful technology that creates a three-dimensional data cube including both spatial and spectral information by acquiring several images at different wavelengths across the electromagnetic spectrum. However, the analysis of hyperspectral images poses difficulties due to the substantial volume of data present in the multiple channels of the HSI cube formed.

The utilization of CNNs is well suited for hyperspectral image analysis due to their capacity to autonomously acquire spatial attributes and accomplish the precise classification of hyperspectral data. Through the utilization of convolutional layers, CNNs can extract spatial features from the hyperspectral image, with pooling layers aiding in reducing the dimensionality of these extracted features. This process empowers the network to effectively learn the spatial characteristics and achieve accurate classification results for the hyperspectral data.

CNNs have been used to perform a variety of hyperspectral image-processing tasks, such as segmentation, detection, and classification. For example, CNNs have been used for land-cover classification, where they can accurately classify different land-cover types based on their spectral signatures [82,83]. CNNs have also been used for target detection, where they can detect targets such as mines or vehicles in a hyperspectral image [84,85]. CNNs have also been utilized for hyperspectral image segmentation, which divides the image into various sections according to its spectral and spatial characteristics.

Given the high dimensionality of HSI images, methods that work by utilizing both spatial and spectral data into a single-CNN architecture lead to better results as compared to the architectures that utilize either spatial or spectral dimensions. Such techniques have been implemented for HSI and Lidar datasets, thus utilizing the full potential of HSI and LiDAR data and providing improved results [86,87,88,89]. Different techniques combine spatial and spectral information in a single configuration using feature extraction from many sources at different levels and are used by single or multi-stream CNNs to achieve robust feature extraction. Various approaches involve two-channel CNNs where a 2D CNN focuses on spatial features and 1D CNN focuses on spectral features that are then cascaded at different levels through a cascade network. Similarly, a two-stream CNN with each stream processing data of a different type from a different source and then fusing them in the final convolution layer has also been implemented for land-cover classification through HSI images [72].

**Table 4:** Deep Learning Methods for HSI Images.

Architecture Details	Nature of Dataset	Studies
*Convolutional Neural Network (CNN)*		
Sixty-three images of the City of San Francisco from a custom dataset that was gathered using Google Earth	Satellite Images	Chen et al. [82]
Sixty-three images of the City of San Francisco from a custom dataset that was gathered using Google Earth	Satellite Images	Chen et al. [83]
Custom dataset of 25 hyperspectral images of the porcine eye cornea	Porcine eye cornea images	Noor et al. [84]
Indian Pines & Salinas Valley Dataset	Satellite Images	Yang et al. [85]
Houston & Trento Dataset	Satellite Images	Rasti et al. [86]
Houston & Trento Dataset	Satellite Images	Li et al. [87]
Houston Dataset	Satellite Images	Feng et al. [88]
ICVL & CAVE Dataset	Street Scene Images	Chang et al. [90]
Kennedy Space Center, Indian Pines, Pavia University, Salinas Scene datasets are used to evaluate the proposed DL architecture	Satellite & Urban Images	Luo et al. [91]
Kennedy Space Center, Indian Pines, Pavia University, Salinas Scene datasets are used to evaluate the proposed DL architecture	Satellite & Urban Images	Chen et al. [92]
Custom Diseased Leaves Dataset	Diseased Leaves Images	Liu et al. [93]
Indian Pines, University of Pavia, WHU-Hi-HongHu dataset	Satellite Images	Dong et al. [94]
*Autoencoder-Decoder (AED) Architecture*		
Kennedy Space Center & University of Pavia Datasets	Satellite & Urban Images	Lin et al. [95]
Indian Pines, Pavia University, Salinas Scene datasets are used to evaluate the proposed DL architecture	Satellite & Urban Images	Shi et al. [96]
Indian Pines & KSC datasets	Satellite & Urban Images	Zhao et al. [97]
Indian Pines, Pavia University & Salinas Scene dataset	Satellite & Urban Images	Dou et al. [98]
Pavia University, Indian Pines, Salinas Scenes dataset	Satellite & Urban images	Zhou et al. [75]
Indian Pines, Salinas Scenes, Houston datasets	Satellite & Urban Images	Patel et al. [99]
*Generative Adversarial Networks (GANs)*		
Indian Pines & Pavia University datasets	Satellite & Urban Images	Zhong et al. [100]
Salinas Valley, Pavia University, KSC dataset	Satellite & Urban Images	Zhu et al. [101]
Houston, Indian Pines, Xuzhou Dataset	Satellite Images	He et al. [102]
Indian Pines, Houston2013, Houston2018 dataset	Satellite & Urban Images	Hang et al. [103]
*Recurrent Neural Networks (RNNs)*		
Indian Pines, Pavia University, Salinas Scenes dataset	Satellite & Urban Images	Zhang et al. [104]
Indian Pines & Pavia University dataset	Satellite Images	Hang et al. [105]
Houston, Indian Pines & Pavia University dataset	Satellite & Urban Images	Mou et al. [106]
Indian Pines, Pavia center scene & Pavia University dataset	Satellite & Urban Images	Shi et al. [107]
Indian Pines, Big Indian Pines & Salinas Valley dataset	Satellite & Urban Images	Paoletti et al. [108]

Chang et al. implemented a deep denoising CNN for HSI image restoration in which the learned filters extract spatial information and the spectral data are learned and extracted through the multiple channels of the 2-D filters without distorting the features of the original HSI image and removing different distributions of noise [90].

A CNN-based architecture was proposed by Luo et al. to extract spatial–spectral properties from the target pixel and its surrounding pixels. The extracted features are then transformed into one-dimensional feature maps using convolutions, which are stacked into a 2D matrix to serve as the input image for a standard CNN. The researchers implemented two variations of this network, one incorporating XGBoost and the other utilizing the MNIST model. They conducted performance evaluations on benchmarked hyperspectral image (HSI) datasets, including Kennedy Space Center (KSC), Indian Pines (IP), Pavia University, and Salinas Scenes. The results showed improved accuracy compared to existing CNN architectures commonly used as benchmarks [91].

In order to enhance the performance of HSI classification, Chen et al. implemented 1D Auto-CNNs and 3D Auto-CNNs as spectral-spatial classifiers. They also chose the appropriate number of operations for convolution, pooling, identity, batch normalization, activation function selection, architecture selection, and regularization techniques. The model is then evaluated on the Kennedy Space Center (KSC) dataset, the Indian Pines (IP), Pavia University dataset, and the Salinas Scenes datasets, resulting in better outcomes compared to cutting-edge deep CNN models [92].

Lu et al. have implemented a 3D atrous denoising CNN architecture for HSI images that extracts feature maps both spatially and spectrally, and the proposed multi-branch architecture leads to less training issues, less overfitting risk and texture preservation, leading to the removal of photon and thermal noise. The proposed architecture’s results are superior to those of modern denoising architectures [93].

To improve the categorization of hyperspectral images (HSIs), Dong et al. have put out a brand-new method dubbed Weighted Feature Fusion of Convolutional Neural Network and Graph Attention Network. To increase accuracy, they make use of the capabilities of graph neural networks (GNN) and graph attention networks (GATs). A graph attention network (GAT) is first put into practice using an encoder–decoder design. It then includes an attention mechanism in a convolutional neural network (CNN). The features that were extracted from both networks are then fused together after being given weights. The suggested approach outperforms current state-of-the-art models in the area of HSI classification, according to thorough testing and evaluation [94].

In conclusion, CNNs are an effective tool for hyperspectral image processing and may be applied to a variety of tasks, such as segmentation, detection, and classification.

#### 4.2.2. Autoencoder–Decoder Architectures for HSI Data

An autoencoder is a neural network used for unsupervised learning, comprising an encoder and a decoder. This network architecture collaboratively learns a condensed representation of the input data. The encoder reduces the dimensions of the input data, while the decoder uses this compressed representation to recreate the original data.

In the domain of hyperspectral image analysis, autoencoders offer a valuable technique for acquiring a hyperspectral image representation in low dimensions. The process involves the encoder component taking a high-dimensional hyperspectral image as input and creating an image’s compressed version in a smaller-dimensional space. Subsequently, the decoder component reconstructs the original image using this compressed representation.

The autoencoder undergoes training using a collection of hyperspectral images, attempting to keep the difference between the original image and the reconstruction as small as possible. Following the training phase, the autoencoder becomes capable of compressing novel hyperspectral images into a condensed representation of lower dimensionality. This compressed representation finds utility in diverse applications, including image classification, anomaly detection, and target detection.

Due to their inherent high dimensionality and redundant information, autoencoders are used in hyperspectral image analysis. The rationale behind using autoencoders is to acquire a condensed, hyperspectral image represented in reduced dimensions. This enables a reduction in computational complexity during subsequent analysis tasks while still retaining the vital information within the data.

Lin et al. discussed a framework for spectral–spatial feature extraction through the autoencoder–decoder (AED) architecture. They implemented PCA on the spectral dimension and autoencoder-decoder (AED) architecture in the spatial dimension to extract features for classification. Representations are computed via autoencoders and then passed on to the classifier, and for spatial dimensions, PCA is implemented while keeping the first three principal components. The representations from the AED and the principal components from PCA are further passed through the training AED layers and a final logistic regression layer [95].

Shi et al. have developed a classification framework for hyperspectral image (HSI) analysis that leverages the concept of spectral–spatial features extracted from multi-scale super-pixels using recurrent neural networks (RNN) and stacked autoencoders. The approach involves segmenting the HSI image into shape-adaptive regions using multi-scale super-pixels, which capture object information with enhanced accuracy. By incorporating the super-pixel-based method, the algorithm effectively captures features at various scales and utilizes the RNN architecture to account for feature correlations across different scales [96].

Zhao et al. have introduced a methodology that involves measuring hyperspectral images (HSI) of suitable spatial resolution using pixel-based transformations and class separability criteria. The next step is a hierarchical learning architecture that combines a random forest classifier and a deep-stacked sparse autoencoder. This method’s goal is to use high-level, abstract feature representations that include both spatial and spectral data. The architecture achieves a favorable balance between generalization, prediction accuracy, and operational speed. The researchers conducted experiments on the Indian Pines and Kennedy Space Center datasets, resulting in competitive, state-of-the-art outcomes [97].

Dou et al. developed a band-selection technique for hyperspectral image (HSI) datasets, aiming to reduce redundancy while preserving the original content of the dataset. The HSI data samples are recreated using a small number of useful bands for each pixel in their method, which considers band selection as a challenge of spectral reconstruction. The non-linear inter-dependencies between bands are modeled using an attention-based autoencoder. The autoencoder uses the most informative bands found for each pixel using the attention module to rebuild the raw HSI. By grouping the column vectors of the obtained attention mask and choosing the most representative band for each cluster, the final band selection is accomplished. This approach enables the learning of global non-linear correlations among the bands. The suggested algorithm uses the stochastic gradient descent algorithm to jointly optimize all parameters. The architecture is evaluated on HSI datasets such as Indian Pines, Pavia University, and Salinas, yielding promising and encouraging results [98].

Zhou et al. have tackled the challenge of achieving both low intra-class scatter and large inter-class separation to effectively learn a low-dimensional feature space for hyperspectral image (HSI) classification. They propose a novel architecture called the compact and discriminative stacked autoencoder (CDSAE), consisting of two stages that progressively optimize the feature mappings. In the first stage, Fischer Discriminant Regularization is applied to the hidden layers of the stacked autoencoder. This regularization encourages the network to map pixels belonging to the same class closer together while minimizing reconstruction errors during training. The feature representations obtained from this stage, which include the Fischer discriminant regularization, are then used by the classifier. To prevent the network from becoming excessively deep, the authors employ diverse regularization techniques on the hidden neurons. The University of Pavia, Indian Pines, and Salinas datasets are used to assess the proposed model’s performance in HSI categorization [75].

Patel et al. have proposed an autoencoder based on the CNN architecture for HSI classification, keeping in consideration the huge volume of hyperspectral picture data and limited spatial resolution. By utilizing autoencoders in the initial layers, hyperspectral feature augmentation allows for the acquisition of optimized weights in the initial layers. Therefore, using this approach, features are extracted from the pre-processed HSI data and employed by the classifier via a shallow CNN architecture. The method has since been evaluated on the Indian Pines, Pavia University, Salinas Scene, and Houston datasets, with encouraging results showing that it is comparable with state-of-the-art methods [99].

#### 4.2.3. Generative Adversarial Neural Networks (GANs) for HSI Data

Generative Adversarial Networks (GANs) are a specific class of deep neural network architectures that prove highly advantageous in generating new data samples closely resembling an existing dataset. In the field of hyperspectral image analysis, GANs offer the potential to generate novel hyperspectral images that exhibit similarities to the original dataset. This capability can be harnessed for diverse purposes such as data augmentation, denoising, and other relevant applications.

GANs operate on the principle of training two deep neural networks concurrently: a generator network and a discriminator network. The discriminator network takes a sample as input and distinguishes between actual and generated/fake samples, whereas the generator network takes a random noise vector as input and generates a new sample. During training, the generator network gains the ability to produce samples that closely resemble genuine samples, making it harder for the discriminator network to tell them apart. Simultaneously, the discriminator network learns to distinguish between genuine and artificial data with accuracy.

In the context of hyperspectral image analysis, the generator network within GANs can be trained to generate novel hyperspectral images that exhibit similarity to the original dataset. Conversely, it is possible to train the discriminator network to distinguish between actual hyperspectral images and those produced by the generator. Once the GAN is trained, the generator network becomes capable of producing new hyperspectral images, which can be utilized for various purposes, including data augmentation or other relevant applications.

Some of the challenges of using GANs in hyperspectral image analysis are as follows.

1. Data dimensionality: Hyperspectral data typically have high-dimensional feature spaces, which can make training GANs more complex and computationally demanding. The increased dimensionality can lead to difficulties in capturing the intricate distributions and correlations present in hyperspectral data.
2. Limited training data: GANs often require a large number of training data to effectively learn and generate high-quality samples. However, collecting and labeling large-scale hyperspectral datasets can be expensive and time-consuming, resulting in limited training data availability for GAN models.
3. Mode collapse: Mode collapse refers to a situation where the generator network fails to capture the full diversity of the target hyperspectral data distribution and instead produces only a limited set of samples. This can result in generated hyperspectral images that lack variability and fail to represent the entire data distribution.
4. Evaluation and validation: Assessing the quality and performance of GAN-generated hyperspectral data can be challenging. Metrics and evaluation methods specific to hyperspectral data need to be developed to ensure the generated samples are accurate representations of the original data and satisfy domain-specific requirements.
5. Sensitivity to noise and artifacts: GANs can be sensitive to noise and artifacts present in hyperspectral data. This noise and artifacts can affect the training process and influence the quality of the generated samples, requiring additional preprocessing steps or regularization techniques to mitigate their impact.
  Addressing these challenges and developing robust GAN architectures tailored for hyperspectral data analysis can lead to improved generation and utilization of synthetic hyperspectral data for various applications.

To address the classification of hyperspectral images (HSIs), Zhong et al. have suggested a system that combines a generative adversarial network (GAN) with a conditional random field (CRF). This approach combines a probabilistic graph model and semi-supervised deep learning. By employing convolutions and transposed convolutions, the authors extract discriminative features from labeled HSI samples. These features are then used as input to GANs, which generate additional labeled HSI samples. This approach addresses the issue of limited labeled training data. Dense CRFs are constructed using random variables initialized with the softmax predictions from the GANs. The CRFs are further fine-tuned on the HSI data to obtain refined classification maps [100].

Zhu et al. implemented a CNN and a GAN for fake image classification. They implemented a 1D GAN for spectral classification and a 3D GAN for spectral–spatial classification. The CNN and GANs are trained concurrently, and the GAN distinguishes between the fake data and the genuine data by creating false inputs from the original data. Training both together fine-tunes the discriminative CNN, increases the generalization capability of the discriminative CNN, and also solves the issue of fewer original training samples. The proposed pipeline is tested on Salinas, Indian Pines, and Kennedy Space Center dataset [101].

He et al. have tackled the challenge of class imbalance in GANs, where the minority class is often associated with a fake label. To overcome this issue, they have introduced a semi-supervised GAN that incorporates a transformer architecture. This transformer is semi-supervised to prevent self-contradiction during classification and discrimination tasks. The generator, equipped with skip connections, is responsible for generating HSI patches, while, to prevent the loss of critical information during the generating process, the transformer captures the semantic context. The generalization capability of the transformer is enhanced through data-augmentation techniques. On the Houston, Indian Pines, and Xuzhou datasets, the suggested model is assessed, and it exhibits competitive classification performance when compared to leading-edge models [102].

By using the vast quantity of information included in unlabelled HSI data, Hang et al. suggested a multitask generative adversarial network (MTGAN) to cope with the issue of the poor availability of HSI training examples. The generator network reconstructs the HSI cube, including the labeled and unlabelled areas, and then attempts to recognize the class of the cube through its classification module. The cube coming from the reconstructed data and the original data is distinguished using a discriminator network, whose generalizability is improved as it receives the original input samples and the nearly original reconstructed samples from the generator. Skip connections are implemented in the generator, and the classifier module to make use of information in the shallow layers. The outcomes of the suggested architecture are compared to those of the state of the art in [103].

#### 4.2.4. Recurrent Neural Networks (RNN) for HSI Data

RNNs, a specific type of neural network, are suitable for handling sequential data processing tasks. When applied in hyperspectral image analysis, RNNs prove beneficial in processing the sequential information embedded within hyperspectral images.

Hyperspectral images capture the spectral response of each pixel across numerous wavelengths, forming a sequence of spectral bands. RNNs can efficiently handle these sequences by extracting useful features that may be used in a variety of hyperspectral image analysis applications, such as classification. Using an RNN known as a Long Short-Term Memory (LSTM) network is one such strategy. The vanishing gradient issue that might arise when using standard RNNs to process lengthy data sequences is addressed by LSTMs. Within hyperspectral image analysis, the application of LSTM networks involves handling input sequences consisting of spectral bands. Each band represents the spectral response of a pixel at a specific wavelength. The LSTM network processes this sequential data, enabling the extraction of features that encapsulate both spatial and spectral attributes present in the image. Then, these characteristics may be applied to categorization or other tasks suc as target or anomaly detection. A prominent area of study in the domain of remote sensing is the application of RNNs in hyperspectral image processing, which has demonstrated promising results.

In order to analyze hyperspectral pixels as sequential data and fill in any missing categories, Mou et al. have introduced a neural network architecture based on RNNs. In contrast to conventional rectified linear units or tanh, the authors’ method uses the parametric rectified tanh (PRetanh) activation function, which permits the use of larger learning rates without worrying about divergence during training. To enhance the efficiency of processing hyperspectral data and reduce parameter complexity, a modified gated unit is employed in the recurrent layer. Three aerial hyperspectral image datasets are used to assess the proposed model, producing results that are competitive in terms of performance [106].

To use redundant and supplementary information seen in hyperspectral images (HSIs), Hang et al. devised a cascaded RNN architecture using gated recurrent units. The suggested model has two layers, the first of which seeks to remove duplicate information between neighboring spectral bands and the second of which aims to collect supplementary information from non-adjacent HSI bands. An integrated convolutional module with convolutional layers handles the spectral–spatial properties of the HSI data. Two HSI datasets are used to test the model’s performance, and the results are not as competitive as those from other current models [105].

Zhang et al. have devised an RNN-based approach, known as the local spatial sequential (LSS) method, to effectively extract both local and semantic information from hyperspectral images. In order to create LSS features, the approach first extracts low-level data such as texture and morphological profiles. The RNN architecture is then given these LSS characteristics for additional processing. The RNN generates high-level features, which are then passed through a softmax layer for final classification. To further enhance performance, the researchers introduce a non-local spatial sequential method into the RNN architecture, referred to as NLSS-RNN. This method identifies non-local similar structures and incorporates their information into the LSS features, preserving spatial information and integrating knowledge from non-local similar samples. The suggested method is tested on several HSI datasets and produces competitive results that exceed cutting-edge techniques [104].

In order to explicitly address the issue of high dimensionality in HSI data, Paoletti et al. have developed an enhanced RNN architecture for hyperspectral image (HSI) classification. Recognizing that conventional RNNs struggle to handle large datasets and high-dimensional input, the researchers designed a modified RNN with simpler recurrent units. This enhanced architecture not only offers competitive classification accuracy but also demonstrates improved scalability when dealing with HSI data [108].

Recurrent neural networks (RNNs) have been used by Shi et al. to offer a novel framework for hyperspectral image (HSI) classification that captures the spatial dependence and spectral–spatial characteristics of nearby pixels. They suggest multi-scale hierarchical recurrent neural networks (MHRNNs) to circumvent RNNs’ restriction to processing just 1D sequences. They create multi-scale image patches of the focal and peri-focal pixels to carry out this method, and then they use 3D convolutional neural networks (CNNs) to extract local spectral–spatial information. The spatial dependency at various scales is then captured by building multi-scale 1D sequences based on the 3D local spectral–spatial properties and employing MHRNNs. In order to produce competitive visual results and classification accuracy, the suggested architecture takes into account both local spectral–spatial properties and the spatial dependence of non-adjacent picture patches. Performance evaluation demonstrates its state-of-the-art performance [107].

## 5. Evaluation Metrics for Deep Learning HSI Data Analysis

Deep learning architecture’s performance evaluation is influenced by a number of variables, including the architecture itself, the qualities of the data, and the issue being addressed. Different evaluation criteria are used for different architectures. Typical performance metrics include *Peak Signal-to-Noise Ratio (PSNR), Structural Similarity Index Measurement (SSIM), and Spectral Angle Mapping (SAM)*. *Overall Accuracy (OA)*, which calculates the average accuracy across all classes, *Average Accuracy (AA)*, which calculates the average accuracy across all classes, and *Kappa Coefficient (kappa)*, which is a statistical measure of agreement between the classification map and the ground truth, are also frequently used performance metrics. These measures aid in evaluating the effectiveness and caliber of deep learning models across many domains.

### 5.1. Structural Similarity Index Measurement (SSIM)

Based on the premise that the Human Visual System (HVS) model may be modified to extract structural elements from a scene, the Structural Similarity Index Measurement (SSIM) was developed. It consists of three basic local comparison functions with their comparison constants, i.e., the luminance comparison function, dependent on the constant $C_{1}$ computed through (7); contrast comparison function, dependent on the constant $C_{2}$ computed through (8); and the structure comparison, function dependent on the constant $C_{3}$ computed through (9) [109]

(7) $$luminance\left(a,b\right)=\frac{2\mu_{a}\mu_{b}+C_{1}}{\mu_{a}^{2}+\mu_{b}^{2}+C_{1}}$$

(8) $$contrast\left(a,b\right)=\frac{2\sigma_{a}\sigma_{b}+C_{2}}{\sigma_{a}^{2}+\sigma_{b}^{2}+C_{2}}$$

(9) $$structure\left(a,b\right)=\frac{\sigma_{a}b+C_{3}}{\sigma_{a}+\sigma_{b}+C_{3}}$$

where *a* and *b* are two signals being processed, $\mu_{a}$ and $\mu_{b}$ denote the sample means of *a* and *b*, $\sigma_{a}$ and $\sigma_{b}$ are the sample standard deviations of a and *b*; and $\sigma_{ab}$ is the correlation coefficient between *a* and *b*. The constants $C_{1}$, $C_{2}$, and $C_{3}$ discussed above are used as stabilizers for the algorithm when the denominators approach zero [109].

The final output for the SSIM is computed by combining these three comparison functions with α,β and γ describing how the three comparison functions affect SSIM’s ultimate result, which is determined using the formula below [109]

(10) $$SSIM=luminance{\left(a,b\right)}^{\alpha }\cdot contrast{\left(a,b\right)}^{\beta }\cdot structure{\left(a,b\right)}^{\gamma }$$

### 5.2. Peak Signal-to-Noise Ratio (PSNR)

Peak Signal-to-Noise Ratio (PSNR), which is calculated using the formula below, is used as an assessment metric for reconstruction quality in image compression and image denoising [110]:

(11) $$PSNR\left(h,k\right)=10\ln_{10}\frac{255^{2}}{MSE(h,k)}$$

where *h* is the reference grey-level eight-bit image and *k* is the original image, both of size i×j, and where MSE is the mean squared error. Results also reveal that when MSE decreases, PSNR increases, implying that the greater the PSNR number, the higher the image quality. However, small values of PSNR indicate a large numerical difference between images [110].

### 5.3. Spectral Angle Mapping (SAM)

Spectral Angle Mapping (SAM) is an efficient and reliable algorithm used to assess the similarity between image spectra and reference spectra. It is commonly employed for classification purposes and uses the reflectance spectra of picture pixels and reference materials to compare the spectral similarities. Reflectance represents the percentage of light reflected by a specific material across various wavelengths, and SAM quantifies the angle between these spectra to determine their similarity [111,112]. SAM measures the degree of spectral similarity between two spectra by computing the angle between them while treating them as vectors in n-dimensional space. The following formula is used to compute SAM as a dot product between the test spectrum *T* and the reference spectrum *R* [112]

(12) $$\alpha =\cos^{-1}\frac{\sum_{i-1}^{b}t_{i}r_{i}}{{\left(\sum_{i=1}^{b}t_{i}^{2}\right)}^{1/2}{\left(\sum_{i=1}^{b}r_{i}^{2}\right)}^{1/2}}$$

where *b* is the number of bands, $t_{1}$ is the test spectrum, and $r_{1}$ is the reference spectrum [112].

The values for OA and AA would be identical if the number of samples for each category was the same, which is rarely the case in reality. AA is used with OA because OA alone is not a good measure, as some rare categories/classes may be present, which would be ignored; thus, AA is also used as an evaluation measure for classification.

CNN designs have been evaluated using a variety of measures, including overall accuracy (OA), average accuracy (AA), and Kappa coefficients (K). Chen et al. adopted these three criteria for their evaluation process. The classification accuracy was computed as the mean value ± standard deviation, obtained from ten separate runs of experiments using different random training samples [92]. To evaluate the proposed CNN architecture’s classification accuracy, Dong et al. used a thorough set of six assessment criteria. These criteria were training and testing duration, kappa coefficient, per-class accuracy, overall accuracy (OA), average accuracy (AA), and kappa. The reported experimental results were calculated using the average of five separate runs, ensuring both qualitative and quantitative evaluation of the CNN architecture’s performance [94]. According to five commonly used criteria, namely PSNR, SSIM, feature similarity index measurement (FSIM), relative global-dimensional synthesis error (ERGAS), and spectrum angle map (SAM), Liu et al. analyzed the performance of their suggested architecture [93]. Luo et al. used overall accuracy and average accuracy as evaluation metrics [91]. Chang et al. employed several quantitative evaluation metrics, including PSNR, SSIM, ERGAS, and SAM, to assess the quality of their restored images. PSNR and SSIM were utilized for evaluating spatial quality, where higher values indicated better restoration. On the other hand, ERGAS and SAM were used for assessing spectral quality, with lower values indicating improved restoration. By considering these metrics, the authors were able to comprehensively evaluate the performance of their restoration method [90]. Based on classification maps for the HSI branch, LiDAR branch, and their proposed two-branch CNN, Feng et al. assessed their suggested CNN architecture. They evaluated the accuracy assessment based on the OA and kappa coefficient calculated based on test samples [88]. Rasti et al. conducted a comprehensive evaluation of different classification approaches, considering multiple aspects. Using criteria such as total accuracy, the average accuracy, and kappa coefficient, they evaluated the categorization accuracy. To assess the effectiveness of the categorization algorithms, the CPU processing time was also considered. The authors were able to offer a thorough review of the effectiveness and the computational efficiency of the various categorization algorithms by taking these assessment factors into account [86]. Chen et al. conducted an assessment of their proposed architecture by considering three performance metrics: false alarm rate (FAR), precision rate (PR), and recall rate (RR). The PR counts the percentage of successfully recognized cars among all detected objects, whereas the FAR calculates the ratio of false alarms to the total number of vehicles. On the other hand, the RR assesses the proportion of properly identified automobiles to all vehicles in the dataset. The authors were able to analyze the precision and efficiency of their architecture for identifying automobiles by using these criteria [83].

Various evaluation metrics have been implemented for the performance evaluation of autoencoder–decoder-based deep learning architectures for HSI data, some of which are discussed below. Lin et al. first analyze how well the AED architecture reconstructs the input through epochs for which one sample is visualized and analyzed at each epoch, with findings from many hundreds of iterating epochs demonstrating impressive reconstruction. The findings of the evaluation of the classification performance for eight autoencoders with various hidden layer sizes indicate that increasing depth does not aid in lowering the classification rate. The overall test set errors are compared for performance on the two datasets used for experimentation [95]. Shi et al. assessed the classification results by calculating the average values from ten independent experiments. They took into account the kappa coefficient, average accuracy, and total accuracy as assessment criteria. By averaging the results across multiple experiments, the authors obtained a more comprehensive evaluation of the performance of their classification approach [96]. Zhao et al. used the kappa coefficient and overall accuracy as two assessment measures to statistically evaluate the categorization performance of their suggested design. Utilizing the original spectral feature set, the new feature set, and the spectral-spatial feature set, among others, these metrics were utilized to assess the performance of the architecture [97]. Using total accuracy curves, Dou et al. assessed how well their implemented architecture performed. They plotted the values of overall accuracy (OA) against the number of selected bands and also considered the kappa coefficients as an evaluation metric [98]. Zhou et al. evaluated the classification results based on the general accuracy of three HSI classification datasets using spectral vs. spectral-spatial characteristics as inputs to the hidden-layer neurons. The local Fisher discriminant regularization and diversity regularization are measured in terms of the OA, AA, kappa coefficient, and box-plot analysis of the overall accuracy values of different methods [75]. Patel et al. utilized overall accuracy, kappa coefficient, precision, recall rate, and f1 score for classification performance evaluation [99].

Somewhat similar evaluation metrics have been used to analyze performance in GANs for HSI deep learning architectures. Zhong et al. have evaluated GAN-CRF models with their competitors based on the classification accuracy of each land cover class through overall accuracy, average accuracy, and kappa coefficient. Moreover, the training and testing time for all semi-supervised GANs is also recorded to analyze computational complexity [100]. Zhu et al. evaluated their GAN-based deep learning architecture for HSI data through OA, AA, and kappa coefficient by implementing the architecture by randomly selecting data for the training and testing sets for 500 epochs, and the classification results are evaluated in the form of mean ± standard deviation [101]. He et al.’s GAN-based deep architecture for HSI data categorization was also evaluated using the overall accuracy, average accuracy, and kappa coefficient [102]. Hang et al. evaluated the overall accuracy, average accuracy, and kappa coefficient as assessment metrics to evaluate the performance of their network [103].

RNN-based deep learning architectures for HSI data were evaluated using measures that are similar to performance assessment metrics. In addition, Mou et al. implemented the OA, AA, and kappa coefficients to compare their performance to that of the most popular vector-based classification models [106]. Hang et al. evaluated the performance accuracy of their proposed RNN-based deep learning architecture for HSI data through OA, AA, per-class accuracy, and kappa coefficient [105]. For 10 sets of trials, Zhang et al. used the kappa coefficient, average accuracy, and total accuracy as performance indicators [104]. The usual performance evaluation criteria, such as kappa, average accuracy, and total accuracy, were used by Paoletti et al. In order to assess the effectiveness of various implementations; they also logged the runtime and the number of parameters [107].

## 6. Discussion and Conclusions

Challenges in spectral unmixing have to be addressed at higher priority because spectral unmixing is one of the initial steps of HSI analysis. As was already explained, the two processes of spectral unmixing are end-member extraction and abundance estimate. The first problem that arises in end-member extraction is the assumption of end-members in the data. It has to be considered that while working with real scenes there may be no completely pure pixels in the sample. In certain samples, locating pixels with the most pure spectral fingerprints is a problem in and of itself. Another challenge is the initial determination of a number of end members that may not be accurate; supposing that no end members exist in the data, many end-member extraction algorithms would result in inaccurate results because they are based on the signature purity criteria instead of pure spectral signature criteria [23,113]. It is challenging to obtain the same results repeatedly when running end-member extraction methods with the same or different initial parameters due to random initialization and computational cost. FPPI and ATGP, discussed in the previous section, produce the same initial conditions, thus solving the issue of inconsistent results [23].

Some of the major challenges in hyperspectral document image analysis include the following.

1. *Data Volume and Complexity:*
  Hyperspectral document images can contain hundreds or even thousands of spectral bands, leading to large data volumes and complexity. Processing and analyzing such large volumes of data can be computationally intensive and time-consuming.
2. *Preprocessing:*
  Hyperspectral images require significant preprocessing to remove noise, normalize the data, and correct for any artifacts that may be present in the data.
3. *Spectral Variability:*
  The spectral signature of a document can vary depending on factors such as ink type, paper type, and lighting conditions. This variability can make it difficult to develop robust algorithms for document analysis.
4. *Dimensionality Reduction:*
  Given the huge number of spectral bands, dimensionality-reduction techniques are frequently required to simplify the calculation and boost the analysis’s precision.
5. *Spectral Mixing:*
  When analyzing hyperspectral images, it is possible to encounter spectral mixing, where multiple spectral signatures are present in a single pixel or region of interest. This can make it challenging to accurately identify and classify different features in the image.
6. *Limited Availability of Data:*
  The availability of hyperspectral document image datasets is limited, making it challenging to develop and test new algorithms and techniques.
7. *Interpretability:*
  The many spectral bands included in hyperspectral photographs might make it challenging to understand the study’s findings, especially for non-experts.

Overall, hyperspectral document image analysis is a challenging and complex field, requiring expertise in areas such as signal processing, machine learning, and computer vision.

## Figures and Tables

**Table 2:** HSI Python Implementations.

Method	Weblink	Access Date	Reference
*Available Implementations of End-Member Extraction Algorithms*			
Pixel Purity Index (PPI)	PPI Python Implementation WebLink	13 March 2023	Joseph W. Boardman [50]
Fast Iterative Pixel Purity Index (FIPPI)	FIPPI Python Implementation WebLink	13 March 2023	Plaza et al. [51]
Automatic Target Generation Process (ATGP)	ATGP Python Implementation WebLink	13 March 2023	Ren et al. [52]
Vertex Component Analysis (VCA)	VCA Python Implementation WebLink	14 March 2023	Nascimento et al. [53]
N-FINDR	NFINDR Python Implementation WebLink	14 March 2023	Winter et al. [54]
*Available Implementations of Abundance Estimation Methods Algorithms*			
Unconstrained Least Squared Methods (ULS)	ULS Python Implementation WebLink	15 March 2023	Torres et al. [16]
Non-Negative Least Squares (NNLS)	NNLS Python Implementation WebLink	15 March 2023	Torres et al. [16]
Unsupervised Non-Negativity Constrained Least Squared Methods (UNCLS)	UNCLS Python Implementation WebLink	16 March 2023	Torres et al. [16]
Fully Constrained Least Squared Method (UFCLS)	FCLS Python Implementation WebLink	16 March 2023	Heinz et al. [55]
Image Space Reconstruction Algorithm (ISRA)	Python implementation is not available	Not Available	Samuel Rosario Torres [17]
HSI Abundance Estimator Toolbox (HABET)	Python implementation is not available	Not Available	Samuel Rosario Torres [17]

**Table 3:** Methods used in HSI Implementations.

Methods and Nature of Dataset	Authors
Historical document enhancement on historical documents from the National Archief of the Netherlands NAN).	Kim et al. [10]
Acquisition, pre-processing, and implementation review of the HSI data for signature segmentation, forgery detection, ink mismatch analysis, historical document analysis, and study of cultural artifacts	Qureshi et al. [1]
Custom dataset of 300 hyperspectral document images captured at 2.1 nm resolution through a hyperspectral camera.	Butt et al. [9]
A subset of 100 hyperspectral images from the dataset proposed by Malik et al. [9] is utilized for signature extraction.	Iqbal et al. [58]
Review hyperspectral image data from Hyperion, CASI, and Headwall Micro-Hyperspec as well as multispectral images from Landsat, Sentinel 2, and SPOT for agricultural research	Lu et al. [12]
Review of deep learning techniques for agricultural studies on the hyperspectral images from the Indian Pines, Salinas, and University of Pavia datasets.	Wang et al. [13]
Custom HSI-MIR and HSI-NIR pictures with near- and middle-infrared hyperspectral images are connected with projection pursuit and PCA for the investigation of counterfeit documents in forensic situations	Pereira et al. [6]
Review of principles, instrumentation, and analytical techniques for HSI analysis and processing for forensic science applications.	Edelman et al. [7]
A state-of-the-art deep learning network for ink mismatch detection is proposed and tested on the UWA Writing Ink Hyperspectral Images (WIHSI) database for forgery detection.	Khan et al. [11]
Method for soil mineralogical changes detection due to petroleum seepage through multispectral images from Landstat7 and Advanced Land Imager (Ali) and hyperspectral images from EO-1 and Hyperion.	El-Hadidy et al. [8]
A bespoke collection of 1200 ground hyperspectral pictures acquired with the GER 1500 spectroradiometer allows for comparison with geographic surveys, ground hyperspectral data, aerial photography, and high-resolution satellite imaging for archaeological research	Sarris et al. [5]
Hyperspectral object detection and classification network with custom HSI dataset of 400 hyperspectral images for object-level target detection.	Yan et al. [3]
Visual saliency model for salient feature extraction and salient object detection on ground-based HSI datasets collected by Foster et al. [59,60] and Harvard University [61].	Liang et al. [62]
Military object detection using PCA, k-means clustering, and self-similarity was tested on San Diego HSI dataset.	Chen ke. [63]
Comparison of existing performance accuracies of deep learning approaches on Indian Pines, Salinas, Pavia Center, and Kennedy Space Center (KSC) datasets.	Petersson et al. [14]
HSI Classification through contextual CNNs with performance tested on the Indian Pines dataset, Salinas dataset, and Pavia University dataset and compared with bench-marked models	Lee et al. [64]
A systematic review of deep learning-based HSI classification methods available in the literature and a comparison of available strategies.	Li et al. [65]
A bespoke dataset of 60 pictures with 121 channels each collected by an NH series hyperspectral camera, serving as the testing and training ground for a two-stage deep learning hyperspectral neural network for person identification on sea surface.	Lu Yan et al. [15]
